# The generation mechanism of Chinese college students’ classroom silence: a qualitative study based on triadic interaction theory

**DOI:** 10.3389/fpsyg.2026.1746284

**Published:** 2026-01-29

**Authors:** Hairong Shi

**Affiliations:** School of Marxism, Chuzhou University, Chuzhou, China

**Keywords:** Chinese university students, classroom silence, core factors, formation mechanism model, triadic reciprocal determinism

## Abstract

**Introduction:**

Classroom silence among Chinese university students has become a distinctive educational phenomenon, impacting individual student development, university classroom teaching, and the cultivation of innovative talent at the national level, among other aspects. Thus, its underlying mechanisms urgently require further exploration.

**Methods:**

This study adopted qualitative research method, selected 10 subjects with the most information by purposive sampling, collected first-hand data by semi-structured interview, and used the ternary interaction theory as the theoretical analysis framework.The original data were coded by using the software NVivo11.This study adopts a novel perspective based on triadic reciprocal determinism to investigate the core factors and theoretical model underlying the formation of classroom silence among university students.

**Results:**

The research identifies three core dimensions contributing to classroom silence—individual, environmental, and behavioral factors. Individual factors include self-efficacy, silence motivation, and psychological state; environmental factors include teacherrelated influences, peer influences, and collectivist norms; and behavioral factors include the self-reinforcement of silence, environmental adaptation to silence, and behavioral choices regarding silence. Classroom silence progresses through four interconnected stages, namely, the triggering stage, reinforcement stage, solidification stage, and cyclical stage, each reflecting bidirectional interactions and dynamic cycles among individual– environmental–behavioral factors.

**Discussion:**

The theoretical model of classroom silence formation under triadic reciprocal determinism reveals the dynamic and interactive nature of classroom silence, providing a more nuanced understanding of its mechanisms in terms of dynamism, systematicity, and intervention potential. This model holds significant practical value. At the explanatory level, it provides a clear answer to the question “why classroom silence becomes increasingly prevalent,” revealing the “self-reinforcing” logic of silence. At the diagnostic level, it assists teachers in identifying the stage of classroom silence and formulating targeted strategies. At the intervention level, it clarifies the focus of interventions at different stages.

## 1 Introduction

The classroom silence of Chinese college students has become a unique “educational fact” worthy of in-depth investigation and careful study (Lv, 2018). In the actual classroom teaching of colleges and universities, the phenomenon in which college students keep silent in the face of teachers’ classroom questions is widespread, and there are few active respondents, especially those who question the problems that may exist in teachers’ teaching or ask questions to teachers (Zhu et al., 2017). As a scholar once stated, “The general state of depression in Chinese universities is melancholy. The classroom is a place that should arouse brainstorming, but it is terribly quiet” (Zhang, 2007). Why are so many students silent in Chinese university classrooms? What causes their classroom silence?

## 2 Literature review

### 2.1 Classroom silence and its influencing factors

Classroom silence is a typical phenomenon in current classroom teaching and is related to many important fields, such as classroom participation, class construction, and teaching strategies (Chen, 2023). Classroom silence is not only the complete absence of audible speech but also the absence of specific topics or requests from the teacher. Such silence can be interpreted as not only an obedient attitude toward classroom order and respect for teachers but also resistance to institutional power or self-protection based on intellectual incompetence or cultural alienation (Katherine, 2018; Peng et al., 2023). According to the nature of classroom silence, it can be divided into positive silence and negative silence (Juma et al., 2022; Zheng, 2011). Passive silence is particularly prominent among Chinese learners and usually does not lead to better learning outcomes (Jia et al., 2022; Xu et al., 2022).

In recent years, many scholars at home and abroad have focused on the influencing factors and causes of college students’ silence in class and have provided explanations from the following three different research perspectives. First, scholars explain this phenomenon from a cultural perspective. In Oriental culture, especially Confucian culture, it is important for Chinese students to be silent and conservative in the classroom (Lv, 2016). China’s educational philosophy and learning tradition are deeply influenced by Confucian culture. People with Confucian cultural backgrounds tend to be diligent and modest, emphasize the importance of order, respect authority, and be reluctant to speak up and challenge (Bush and Haiyan, 2000). The high sense of power distance brought about by Chinese culture often widens the gap between teachers and students, which makes students reluctant to ask teachers questions for fear of being seen by teachers as a challenge to their authority (Holmes, 2004). Second, scholars explain this phenomenon from the perspective of psychology. College students’ classroom silence comes from their own personality and psychological aspects, and students’ personality traits, motivations, emotions, and psychological states can affect their classroom silence behavior (Lei et al., 2017; Zhang, 2019). Classroom silence is the self-emotional expression of students all the time and includes the anxiety of facing self-ignorance, the fear of self-presentation failure, the resistance to being ridiculed and despised, the dissatisfaction and helplessness of being neglected, and the immersion of curriculum and teacher discourse (Chen, 2018). Third, scholars explain this phenomenon from the perspective of pedagogy. From the perspective of educational influence subjects, teachers, peer groups, family members, and so forth directly or indirectly influence students’ classroom silence behavior. Teachers’ lack of creative personality charm, insufficient attention to students’ “life universe” in the teaching process, neglect of teaching art research, and alienation of the teacher–student relationship are important factors affecting classroom silence (Teng, 2009). Feedback from teachers and peers, the class atmosphere, participation time, the participation of group members, and learning equipment also directly affect the enthusiasm of students in classroom interaction (Muuro et al., 2014). Studies have shown that classroom norms, class size, student structure, teaching content, teaching style, teacher–student relationships, and evaluation methods affect whether students are silent (Frambach et al., 2013; Hu and Li, 2014; Wan, 2021; Zhou et al., 2005).

In terms of the research status at home and abroad, the literature on the influencing factors of college students’ classroom silence is more comprehensive, but the existing research on the relationship between the influencing factors of college students’ classroom silence, especially the interaction path between various factors, is still insufficient. Therefore, clarifying the relationships among the influencing factors of college students’ classroom silence is conducive to developing more effective countermeasures and suggestions for overcoming college students’ classroom silence.

### 2.2 Ternary interaction theory

Triadic interaction theory is an important component of the theoretical system of social cognition, which aims to verify and explain individual behavior and proposes that environmental factors, individual factors, and individual behavior are interrelated and mutually influenced (Bandura, 1989). Among them, the individual factor is in the subject position in the interactive relationship among the environment, individual, and behavior, and the individual’s confidence, attitude, motivation, and way of thinking jointly affect his or her behavior. When individual factors and environmental factors reach a state of dynamic equilibrium, individual behavior occurs (Bandura, 2001). Subject, behavior, and environment form an inseparable whole, and the intensity and mode of interaction between them change under different situations, individuals, and forms of activities (Yin, 2022). The theory of triadic interaction builds a bridge between an individual’s internal cognition and the external environment around behavior, emphasizing the complex causal relationships among individuals, the environment, and behavior, which provides a theoretical perspective and research basis for the study of the generation mechanism of college students’ classroom silence.

The generation mechanism of college students’ classroom silence is complex, and classroom teaching is a process of interaction between teachers and students. Students’ classroom silence is influenced not only by personal perception and psychological factors but also by teachers’ teaching state, teaching environment, and other factors. Existing research explores the influencing factors of classroom silence from students, teachers, the environment, and other aspects, but the relationships among the influencing factors are not clear. From the perspective of triadic interaction theory, this study explores the influencing factors of college students’ classroom silence. By exploring the relationships among influencing factors, this paper analyzes how college students’ classroom silence is generated. In this paper, targeted solutions for overcoming the silence of college students in the teaching process are proposed, which has important practical significance for the cultivation of innovative talent and high-quality teaching practices in colleges and universities.

## 3 Research methods

### 3.1 Research design

In this research, a qualitative research method is adopted. Through face-to-face communication between the researcher and the research object, the researcher uses a variety of data collection methods to explore social phenomena in a natural situation. This is an activity in which the researcher interacts with the research object through their own experience to obtain an explanatory understanding of its behavior and meaning construction (Chen, 2000). This study allows interviewees to open their hearts and discuss their real thoughts about the silence in the classroom to ensure the authenticity of the research materials.

### 3.2 Case selection

Purposive sampling uses limited resources to gather the most effective information, including identifying and selecting informative individuals or groups of participants related to the phenomenon of interest (Palinkas et al., 2015). In accordance with the principle of “purposive sampling,” this study extracts the research objects that can provide the maximum amount of information and looks for the research objects that can provide intensive, rich information as a case (Levitt et al., 2018). In a university in China, the researcher used a purposive sampling method to recruit college students with severe classroom silence as study cases. The recruitment criteria were as follows: (1) full-time college students; (2) severely silent students with little classroom interaction; (3) voluntary participation in the study, clear logic, and barrier-free language communication; and (4) willing to exchange relevant topics of classroom silence, good at introspection and reflection, and willing to elaborate on their own views. Recruitment information was published through the researcher’s working relationship network, including recruitment criteria, research objectives, data confidentiality, and withdrawal at any time without reason. During the release of recruitment information, 112 people signed up. The researchers subsequently conducted the first round of screening according to the following four criteria: (1) 1:1:1:1 balance standard for different grades: one, two, three, and four; (2) 1:1 balance standard for the ratio of males to females; (3) 1:1 balance standard for the ratio of humanities, social sciences, and natural sciences; and (4) the speech description of silence in class being concrete, vivid, and logical. In the first round of screening, 30 research cases were selected from 112 people, including 7 first-grade students. There are 7 students in second grade, 8 in third grade, and 8 in fourth grade; 15 are male, and 15 are female; and 15 are in humanities and social sciences, and 15 in natural sciences. After 30 people were screened out, materials were collected through semistructured interviews. The interview sequence is as follows: 2 people were interviewed from each grade in each round, and the senior grade was circulated to the junior grade (the senior grade had more experience and could provide more available data). Then, we alternated between men and women (excluding gender factor errors) and alternating between majors (excluding professional factor errors). In the first round of interviews with eight researchers, the last two first-grade interviews, M04 and F04, had a large amount of code duplication, and there were little new data. At the beginning of the second round of the interview, when the 9th and 10th interview cases, M05 and F05, were interviewed, the collected data were repeated, and there was no new code. New themes appear, and the code repetition rate is greater than 90%. The researcher checked that all the research dimensions had sufficient data support, and after discussion with three coders and invited one expert to review, the researcher confirmed that there was no information gap. Judging from this, in the data collection process of the 10th interview case, the data reached saturation. The study ended with interviews with 10 research cases, all of whom volunteered to participate in the study. No one quit halfway. Among the students, there were 5 male students (represented by the number M) and 5 female students (represented by the number F); there were 5 students majoring in natural sciences and 5 students majoring in humanities and social sciences. To ensure the privacy of the interviewees and the confidentiality of the data, code names, instead of their real names, were used in the full text (see Table 1).

**Table 1:** The basic information of study cases.

Number	Gender	Grade	Subject
M01	Male	Senior year of college	Humanities and social sciences
M02	Male	Third year of college	Natural science
M03	Male	Second year of college	Humanities and social sciences
M04	Male	freshman year	Natural science
M05	Male	Senior year of college	Humanities and social sciences
F01	Female	Senior year of college	Natural science
F02	Female	Third year of college	Humanities and social sciences
F03	Female	Second year of college	Natural science
F04	Female	freshman year	Humanities and social sciences
F05	Female	Senior year of college	Natural science

### 3.3 Data collection

In this study, semistructured interviews were used to collect first-hand data. Each interview lasted 50–75 min and was recorded with the consent of the interviewees before the interview. Before the formal interviews, this study compiled a series of interview outlines to guide the collection of data and invited two college students to conduct preinterviews to optimize the interview outlines. The interview outline includes a number of open questions, covering a number of factors affecting classroom silence. Under the guidance of the interview outline, the researcher encouraged the interviewees to actively communicate and express their ideas and flexibly adjusted the interview content according to the interview situation. The interviewees freely shared their own experiences and mental journeys related to classroom silence and reflected on the internal and external factors of classroom silence until both sides fully understood the emerging theme. The researcher has rich interview experience, and when he or she was keenly aware of the new problems related to the research topic, he or she would dig more deeply to approach the essence of the phenomenon. After each interview, the recordings were transcribed into text information, and the interview diaries were written promptly, in which the scene and details were recalled and the feelings and scenes of the interview were recorded to pave the way for the next interview. When further collected interview data were repeated with the previous data and no new data appeared, the data were considered saturated, and the data collection process was complete. In the course of the study, we strictly followed research ethics, such as the principles of volunteerism, informed consent, confidentiality, and fairness.

### 3.4 Data analysis

On the day of the end of the interview, the recording was transcribed by the researcher word for word, and all the interview recordings and interview notes were translated into text documents. The collection and collation of the preliminary data were completed, which provided the direction and focus for the next data collection process. In this study, NVivo11 was used to log and code the original data, category analysis was used to identify the common themes of the data, and ternary interaction theory was used as the theoretical analysis framework. The spiral research model was used to classify, deeply analyze, and systematically organize the data, and data analysis was carried out simultaneously by three coders. The three coders are research assistants majoring in psychology, pedagogy, and sociology. They have undergone two rounds of rigorous selection—in the first round of written tests, the research assistants coded a paragraph of text and wrote a memorandum to examine their software application ability, text analysis ability, and mastery of coding methods; and in the second round of interviews, the background knowledge of sociology, psychology and pedagogy was examined to better understand the context and jargon of the interviewees. The researchers subsequently conducted systematic training for the three people so that they could fully grasp the theoretical framework and code book of this study and skillfully operate NVivo11 for coding so that they could complete the initial coding (line-by-line and event-by-event), focus coding (categorize, refine), axis coding (establish category links) and theme refinement, and write high-quality memos. Before formal coding, three coders independently coded the same material of this study and carried out a coding consistency test, with a reliability value of 0.81, to ensure that they fully understood the definition, boundary, and example of each code in the code book. During the coding process, researchers and coders met daily to discuss coding difficulties and new findings. When inconsistent analysis opinions were encountered, an open attitude was maintained, differences were resolved through discussion to reach consensus, personal views on the data were avoided, the data were faithfully presented during coding, and the consistency and logic of the data analysis were ensured. The extracted open coding, main axis coding, and selection coding were fed back to the interviewees through repeated communication and feedback to ensure that the research results did not deviate from the perspective of the researchers. This approach could truly reflect the views of respondents. In this study, the data analysis strictly followed the qualitative coding norms to refine the three-level coding. First, in the first open coding stage, the interview data were analyzed and conceptualized word for word, the repeated labels were merged, and the first-level concept labels were preliminarily completed after selection. Second, in the second stage of principal axis coding, the deep meaning of the concept was clarified, and the correlation between the concepts of similarity and difference was compared. According to the theme and content, the event logic of the generation of classroom silence was determined, and the category attribution was abstracted. Finally, in the three-level selective coding stage, the relationship structure between categories was further clarified, and selective coding determined the category of “the core element of the generation mechanism of classroom silence,” which was combined with ternary interaction theory to construct an analysis framework for the generation mechanism of college students’ classroom silence; finally, conclusions were drawn (see Table 2 for the specific coding).

**Table 2:** The coding table of core elements of classroom silence generation mechanism in college students.

Three-level selective coding	Secondary spindle coding		First-level open coding	Sample source material
The Core Elements of College Students’ Classroom Silence	Individual factors	Self-efficacy	High self-efficacy	I think expressing new ideas in public makes me feel confident. If my answer has a new idea, I will be the first to stand up and take the initiative to answer.
			Low self-efficacy	I have no confidence in learning this course. I basically do not interact with the teacher in class. I am afraid that the teacher will remember my name and often ask questions. If sometimes I cannot answer or answer wrong, I will feel embarrassed when I think about it.
		Silence motivation	Altruistic motivation	Higher mathematics is more difficult, the teacher often asked the students to understand a knowledge point, I do not quite understand some places and dare not ask questions, I am worried that I did not learn, if I stand up to ask questions, it will delay the learning time of the whole class, will interrupt the teacher’s class ideas, delay the teacher’s class time and class progress.
			Self-serving motivation	If there is extra time for answering questions in class, which is conducive to the final grade evaluation, I will respond positively to the teacher and strive for more points.
		Psychological state	Herd mentality	In class, no matter what questions the teacher asked, no one answered, and I kept silent.
			Defensive psychology	I have been doing well in my class. I have won scholarships for two years in a row, and I have won many awards in peacetime. If I speak actively in class, my classmates will think I am showing off and showing off my knowledge. Therefore, I am generally relatively low-key in class, can not speak as silent as possible, so as not to be excluded by everyone after class, causing jealousy and verbal attacks from individual students.
			The habit of silence	We has always been used to listen to the teacher, the traditional classroom is the teacher said that students listen, gradually formed a habit, used to listen to the teacher cramming, used to keep silent in the classroom.
	Environmental factors	Teacher factor	Personality traits	One of my favorite teachers is very humorous. His lecture is very infectious. There is a lot of laughter in class. Everyone is very happy. Every time I listen carefully and answer questions actively.
			Teaching style	I prefer teachers who teach moral cultivation and rule of law. They respect students’ideas in class. They are very democratic and easy-going. They often combine theory with practice to talk about many interesting cases. They combine boring knowledge points with students’ actual life, which is easy to understand.
			Teaching ability	The teacher of our food standard class will set up interactive questioning sessions with different difficulties. The students who answer the questions will be praised in time. If they cannot answer the questions, they will not make a fool of us. The teacher will smile and give the answers in time until they help us solve the difficulties.
		Peer factor	Active Peers	When I saw the excellent students speak, I felt both admiration and special pressure. I admire them for their rich knowledge and strong expression ability, and their ability to speak so thoroughly. Stress is worrying that you are not prepared well enough, or that your ideas are not unique enough, and that you are not as good as them in this respect. Due to various concerns, I will give up the opportunity to speak and keep silent.
			Passive and quiet peers	When the very passive and quiet classmate kept silent, I felt a little heavy, as if there was an invisible force suppressing everyone, so that the original lively classroom became quiet in an instant. Everyone is trapped by this silent force and dare not break it easily.
		Collectivist norms	Avoid conflict	In the class group discussion, the teacher raised an open question and asked everyone to express their views freely. I kept silent all the time because my point of view was different from theirs. I am afraid that my speech will be different, that it will lead to group arguments or conflicts, that it will lead to group members’ disgust or dissatisfaction, and that it will ultimately affect group harmony.
			Obey authority	Last week in class, when the teacher pointed out that a classmate’s answer was wrong and needed to be supplemented, no one stood up to supplement. Teachers have profound professional knowledge and are very authoritative in the industry. Everyone is worried that their speeches are not mature enough, so they choose to remain silent to show respect.
	Behavioral factors	The self-reinforcement of silence	Negative feedback from the teacher	Once, I made a point in class, and the teacher said that my point was wrong. I felt embarrassed in an instant, lowered my head subconsciously, feeling “lost a lot of face,” full of self-doubt, and never wanted to speak again.
			Negative feedback from classmates	After my speech, the students next to me laughed and joked, ‘How can you have such an idea? This view is really strange.’. I felt very embarrassed and hurt and blamed myself in my heart for why I had to speak. Later, I felt anxious about speaking in class and tried to keep silent.
		Silent environmental adaptation	An active environment for speaking	The same teacher, the same class content, the first two classes interact very well, the classroom atmosphere is excellent, students are rushing to speak, very happy to express their views, the answers complement each other to make everyone learn more deeply.
			Quiet and depressed environment to maintain silence	But our two classes asked one without saying a word, and the students did everything below, and the teacher was also very helpless. The two classes are not active, lifeless, there is no active person to stand up.
		The choice of silent behavior	Silence	On controversial topics, silence allows me to avoid disagreement or conflict with others and maintain the harmony of interpersonal relationships. When I benefit from silence in class, I will keep it up.
			Make a speech	Classroom silence makes teachers and classmates think that I lack interest or ability, which forms a negative evaluation that I do not actively participate in classroom learning. What’s worse is that the long silence makes me afraid of speaking, even doubt my ability and lose self-confidence in learning. In the future, I will try to overcome the fear of speaking in class and gradually build up self-confidence to speak actively.

## 4 Research results

According to the analytical framework of ternary interaction theory, individuals, the environment, and behavior are interrelated, emphasizing not only the influence and shaping of the external environment on the individual and his or her behavior but also the important decisive role of the individual himself or herself in his or her external behavior and environment. Moreover, this framework reflects the improvement effect of individual behavior on itself and the environment (Bandura, 1989). Therefore, classroom silence is the result of interactions among individuals, the environment, and the behavior of college students.

### 4.1 Core elements of classroom silence generation under triadic interaction theory

#### 4.1.1 Individual factors: the endogenous drive of silence generation

In this study, low self-efficacy, altruistic or egoistic silence motivation, and psychological effects (herd mentality, defense psychology, and silence habits) positively affect the occurrence of classroom silence behavior to varying degrees.

##### 4.1.1.1 Influence of self-efficacy

Self-efficacy is among the key psychological factors of triadic transactional theory, which refers to personal beliefs about the ability to perform specific actions or behavioral processes (Lent and Brown, 2006). Self-efficacy can be assessed by means of task-specific self-efficacy, process efficacy, and obstacle-specific coping efficacy (Lent et al., 2013). In terms of classroom learning, respondents with high self-efficacy take the initiative to speak, while respondents with low self-efficacy are not confident enough to answer the teacher’s questions and dare not speak. The latter type is hesitant about whether their answers are correct or not and whether their answers are perfect or not, and they imagine how embarrassing it would be if the teacher were to often ask them questions and they were to not know the answers or their answers were wrong. “I feel confident in expressing new ideas in public.” “If my answer has a new point of view, then I will be the first to stand up and take the initiative to answer” (M03). “If I only know a little about the teacher’s question, I am not confident about the answer, and I am not sure whether the answer is correct, I will be very hesitant, and it is easy to miss the opportunity to answer the question” (F02). “Our professional teacher once asked a question. I met the teacher’s eyes, and the teacher looked at me expectantly, hoping that I could take the initiative to answer. Questions happen to be what I often learn recently, but I worry that my answers are not comprehensive enough or not brilliant enough, so I silently bow my head and pretend to think hard about the answers “(M04); “I have no confidence in learning this course; I basically do not interact with the teacher in class; I am afraid that the teacher will remember my name and often ask questions. If sometimes I cannot answer or answer wrong, it’s embarrassing to think about such a scene” (F03).

##### 4.1.1.2 Influence of silence motivation

Motivation is an internal process that provides energy and direction for human behavior (Burkley and Burkley, 2020). When students have the knowledge base and expression ability required to participate in the classroom, they consciously retain their own views out of some motivation and consciously choose not to participate in classroom interaction, which is referred to as “motivated silence” by some scholars (Zhang, 2019). In this study, motivational silence, including altruistic silence, the motivation to worry about teachers and classmates, egoistic silence, and the motivation to consider self-interest, often occurs. “In a report and exchange class on the basis of moral cultivation and the rule of law, the students are particularly well prepared to report their learning results, and I am also well prepared, but the class time is not enough.” “There are still many people who have not communicated. To give other students a chance to show, I gave up reporting and kept silent, listened carefully to their learning results, and thought deeply about where my shortcomings were” (F04). “Higher mathematics is more difficult, teachers often ask students if they understand a knowledge point, I do not quite understand some places and dare not ask questions, and I’m afraid it’s just that I did not learn it myself. If I stand up to ask questions, it will delay the study time of the whole class, interrupt the teacher’s thinking in class, and delay the teacher’s time and progress in class” (M04). “If the time for answering questions in class is increased, it is conducive to the final grade evaluation, and I will respond positively to the teacher and strive for more points” (M05). “Once, the teacher talked about a concept that was inconsistent with the views of the authoritative scholars I saw.” “At that time, I thought maybe the teacher was wrong, but I did not question the teacher’s point of view in class. I was afraid that the teacher would make a fool of himself in public. I was afraid that the teacher would think that I was challenging his authority and disrespecting him, which would make the teacher angry and be detrimental to my exam results” (F02).

##### 4.1.1.3 Influence of psychological state

Almost all the interviewees in this study realized that speaking in class can promote learning. Speaking can not only enliven the classroom atmosphere, help teachers understand students’ learning situations, and strengthen communication between teachers and students but also promote students’ concentration, improve students’ language expression ability, promote students’ deep learning, and so forth. Even so, students remain silent about speaking in class. Such behaviors can be classified into three psychological states: herd mentality, defensive mentality, and silence habit. First, herd mentality shows that the respondents follow the crowd, do not like to be in the limelight, and synchronize with their peers. “In class, no matter what questions the teacher asks, everyone does not answer, and I keep silent” (F05). “Sometimes, the teacher’s questions are very simple, but everyone will not say; I say it seems that I like to show off. I love to show off my cleverness, so I follow the crowd and do not speak” (M03). “Four students in our dormitory often sit together in class, and they do not like to answer questions. To keep pace with everyone, I seldom speak in class, so as not to be isolated by them after class” (F04). Second, defensive psychology is characterized by the face-saving and jealousy of others. “I am the class commissary in charge of studies.” “My classmates all think that I am good at this professional course. If I answer wrong, everyone may laugh at me for learning. I am also afraid to see other people’s strange eyes. It’s too humiliating.” (M02). “I have been doing well in my class. I have won scholarships for two consecutive years and won many awards at ordinary times. If I speak actively in class, my classmates will think I am showing off and showing my knowledge. Therefore, I usually keep a low profile in class and try to keep silent if I cannot speak, so as not to be excluded by everyone after class and cause jealousy and verbal attacks from individual students” (F05). Finally, the habit of silence manifests itself as learned classroom behavior. “When I was in high school, the progress of the course was very fast, the time was tight, and the teacher seldom asked the students to ask questions.” “If some students put forward their own ideas in class, teachers generally ignore them and sometimes criticize us for wasting time. After a long time, students develop the habit of silence and are reluctant to speak. Now that I am in college, I still have the same old habit, and I seldom actively respond to the teacher” (M01). “I have always been used to listening to the teacher, and the traditional classroom is that in which the teacher talks and the students listen and gradually forms a habit of listening to the teacher’s cramming and keeping silent in class “(F03).

#### 4.1.2 Environmental factors: exogenous catalysis of silencing

In this study, the teacher’s humorous and optimistic personality traits, flexible and diverse teaching styles, and excellent teaching ability reduce the occurrence of classroom silence so that students have better classroom speech performance; the influence of peers on classroom silence is more complex, and there is a “double-edged sword” effect for positive speaking peers and a “silence contagion” effect for negative quiet peers. Collectivist norms significantly increase classroom silence.

##### 4.1.2.1 Influence of teacher factors

Teachers’ personality traits, teaching styles, and teaching ability affect the respondents’ participation in classroom learning to varying degrees. First, teachers’ personality traits affect classroom silence. Studies have shown that teachers who are cheerful, optimistic, humorous, enthusiastic, and confident have relatively high levels of participation in class (Teng, 2009). In this study, “easy-going,” “humorous,” “enthusiastic,” and “optimistic” were repeatedly mentioned, and most of the respondents reported that they preferred the above characteristics of teachers, whose classroom atmosphere was very good, and they were very willing to speak actively. “One of my favorite teachers is very humorous. His lectures are very infectious. There is a lot of laughter in class. Everyone is very happy.” “Every time, I listen carefully and answer questions actively. The teacher is also very easy-going and has an affinity. When I encounter problems that cannot be solved in class, I am very willing to consult him after class” (M05). Second, the teacher’s teaching style affects the degree of classroom silence. Teaching style is “the unique combination and expression of effective and consistent teaching viewpoints, teaching skills, and teaching style gradually formed in the long-term practice of teaching art and is the symbol of the stable state of individualization of teaching art” (Li, 2002). In this study, the teacher’s teaching style has a greater effect on students’ classroom participation. The teaching style of highly effective teachers can better mobilize students’ enthusiasm and participation in the classroom, but general teachers find it difficult to do so. “At the beginning, our ideological and political teachers tried to create a good classroom atmosphere and actively interact with students, but the students were not interested.” “Few students spoke in class. Later, he did not interact with the students. In class, he talked from the beginning to the end according to the courseware, and every time, he was cramming. Every time I go to class, I find a seat in the middle of the classroom that is the easiest to hide and sit down to do my own thing “(M03). “I prefer a teacher who teaches moral cultivation and rule of law, who respects students’ ideas in class, and who is very democratic and easy-going. The class is wonderful. It often combines theory with practice to talk about many interesting cases and combines boring knowledge points with students’ actual lives, which is also highly important” (F02). Third, the teacher’s teaching ability affects the degree of classroom silence. In this study, the interviewees mentioned that teachers’ abilities include professional knowledge, teacher–student interaction skills, and language methods, and so forth. They believe that knowledgeable teachers, good teacher–student interactions, and artistic language attract them to participate in the classroom. First, teachers with profound knowledge are very popular, and students’ investment in curriculum learning increases in such settings. “I really feel that the philosophy teacher is particularly charming with the spirit of poetry and books.” “She did read many books, which made me feel that the intelligent brain was more sexy than a simple appearance. Finally, she ended up talking about Markusky’s one-dimensional man. It warns us that in this materialistic world and the world wrapped by capitalism, we should still maintain independent thinking and self-thinking” (M05). Second, good interactions between teachers and students can awaken students’ positive learning mood. “The teacher of our food standards class often praises our classmates for their earnest style of study in class, he will set up interactive questioning sessions with different difficulties, and the students who answer them will be praised in time.” “If we cannot answer, we will not make a fool of ourselves. The teacher will smile and give us the answer in time once we help solve the dilemma. Near the end of the semester, teachers will take students to make cakes, bread, or other baked foods” (M05). Finally, the artistic classroom language creates a relaxed and pleasant classroom atmosphere. “For me, as long as the teacher’s lecture is interesting, the atmosphere is relaxed.” “It does not matter whether it is water or not, as long as you think it is meaningful in class. There are many kinds of meaningfulness, such as being able to show one’s own point of view, being able to discuss experiences that are related to life and empathy, and having interesting and challenging homework (M03).

##### 4.1.2.2 Influence of peer factors

The effects of peer groups in schools on adolescent development are as important as parental involvement, teacher quality, and class size, which affect educational outcomes (Coleman, 1966). Studies have shown that the impact of class groups on college students’ academic performance is greater than that of dormitory groups in China, which is close to or even greater than that of individual learning ability (Quan, 2015). In this study, peer influence was an important factor in college students’ classroom silence. The interviewees mentioned that the influence of peers who actively spoke on classroom participation had the effect of “being close to the audience” and the effect of “comparative pressure” and that peers who were passive and quiet had the effect of “silence contagion” on classroom participation. First, the “double-edged sword” effect of actively speaking to peers is discussed. “When the first student raised his hand to speak, I thought the first student to speak was so brave that he could seize the opportunity to express himself so quickly. I will also cheer myself up secretly in my heart, thinking that I will perform well next, and strive for better classroom participation and learning input. Moreover, after the first student speaks, more and more students will speak” (F03). “When I see the outstanding students speak, I feel both admiration and special pressure. I admire them for their rich knowledge and strong expression ability and their ability to speak so thoroughly. Pressure is to worry that you are not well prepared or that your ideas are not unique enough, that you are not as good as they are in this respect, and that your impression in the minds of teachers and classmates is inferior. Due to various concerns, I will give up the opportunity to speak and remain silent” (F05). Second, the “silent contagion” effect of passively quiet peers is discussed. In this study, the silent magnetic field and the silent safety zone of negative peers make students remain silent in class. “When the very passive and quiet classmate kept silent, I felt a little heavy, as if there was an invisible force suppressing everyone, so that the original lively classroom became quiet in an instant.” “This silence is like a black hole. Slowly, the enthusiasm of the students around were sucked away, and everyone seemed to be trapped by this silent force and dared not break it easily” (M02). “The students who really wanted to interact with the teacher in the class would take the initiative to sit in the front row, and the students in the front row would generally speak actively. Students who take the initiative to sit by the side and back will not speak, and these areas are recognized as silent ‘safe zones’. In the ‘safe zone’ of silence, I feel very relaxed because I can temporarily avoid the pressure of speaking and the tension of speaking, and at the same time, I can have more time to think and digest what the teacher teaches, which is a way of self-protection in class” (M01).

##### 4.1.2.3 Impact of collectivist norms

In this study, collectivist norms significantly influenced the practice of silence in the classroom by emphasizing conflict avoidance, group harmony, and obedience to authority. In the classroom setting, the interviewees chose to remain silent for fear of disrupting the order of the group and obeying authority. “In the class group discussion, the teacher raised an open question and asked everyone to express their views freely.” “The other students in the group spoke one after another. I kept silent all the time because my point of view was different from theirs. I also want to express my ideas, but I am afraid that my speech will be different, that it will lead to group arguments or conflicts, that it will lead to group members’ disgust or dissatisfaction, and ultimately that it will affect group harmony. In order not to delay the progress of the group task, I finally chose to compromise and hide my personal views silently in my heart” (F04). “Last week in class, when the teacher pointed out that a classmate’s answer was wrong and needed to be supplemented, no one stood up to defend or supplement it. Teachers have profound professional knowledge and are very authoritative in the industry. Everyone is worried that their speeches are not mature enough, so they choose to remain silent to show respect. Perhaps the teacher’s criticism is correct; although I have different opinions and would like to support my classmates, I feel that I cannot change the status quo of group silence. I am more worried that defending my classmates will lead to confrontation with teachers. In order to avoid conflict, I also choose silence like everyone else” (F03).

#### 4.1.3 Behavioral factors: intensifiers of silence generation

Classroom silence refers to college students’ silent behavior in the classroom and the chain feedback caused by the behavior, which is a “dynamic bridge” connecting the individual and the environment. In this study, the self-reinforcement of classroom silence reduces the willingness to speak next time, the environmental adaptation of classroom silence forms the reinforcement of silence at the environmental level, and the behavior choice of classroom silence urges individuals to constantly introspect and reflect.

##### 4.1.3.1 Self-reinforcement of classroom silence

Three interviewees said that they encountered negative feedback from teachers and classmates when they spoke in class. “At that time, they wanted to find a crack in the ground. They were full of embarrassment, shame, anxiety, self-doubt, frustration, and other negative emotions, which led to their loss of interest and enthusiasm in class discussion. They felt anxious about speaking in class and kept silent in class to the maximum extent. “Once, I put forward a point of view in class, and the teacher said that my point of view was wrong. I felt embarrassed for a moment, subconsciously lowered my head, and avoided eye contact with anyone, feeling that I was “losing face” and full of self-doubt. I felt that my expression is not good enough or that my views are not correct, which made me doubt my ability and knowledge level. I thought over and over again whether I was not prepared enough. Is there something wrong with my understanding? This kind of self-doubt reduces my self-confidence and even affects my interest in the course. In the rest of the class, I tried to avoid speaking and keep silent as much as possible so as not to become the focus of attention again” (M02). “After I spoke, the students next to me laughed and said, “How can you have such an idea? This view is really strange. I felt very embarrassed and hurt and blamed myself in my heart for having to speak. Later in class, I felt anxious about speaking in class and tried to remain silent. Even if they have ideas, they dare not express them easily for fear of being laughed at by their classmates again” (F03).

##### 4.1.3.2 Environmental adaptation of classroom silence

This study revealed that active classes make students dare to speak actively in class, while quiet and depressed classes make students afraid of speaking in class, and they are more willing to keep silent and stay in line with their classmates. “There are four classes in one grade of our major, and most of the courses are divided into four classes, which are divided into the first two classes and the last two classes. With the same teacher and the same class content, the first two classes interact very well, the classroom atmosphere is excellent, and they listen very carefully, but our two classes are silent when asked. The students do everything below. The teacher is also very helpless. He often compares us with the first two classes in class. When the four classes have a public class together, they seem to be two worlds, and the contrast is even more obvious. I found that the first two classes were so active that the students rushed to speak. They were very happy to express their views, and the answers complement each other so that we can learn more deeply. Our last two classes were half dead and lifeless, and there were no active people to stand up at all. In fact, I really want to cooperate with the teacher, but everyone is not active; I am not too embarrassed; there is no courage. I also hate this, so I especially envy the first two classes of students” (F02).

##### 4.1.3.3 Behavior choice of classroom silence

The visitor said, “Is it silence or speech? It depends on the pros and cons of classroom silence. If classroom silence brings more certainty benefits, such as avoiding embarrassment and mistakes, avoiding interpersonal conflicts, reducing speech anxiety, and facilitating listening and thinking about other people’s views, visitors will continue to remain silent in class. If classroom silence causes visitors to face more costs, such as the loss of classroom participation opportunities, with more negative comments and a loss of self-confidence in learning, visitors will gradually break the silence in the classroom. “When the teacher asked a question that I did not fully understand, I chose to remain silent to reduce possible mistakes and embarrassment in my speech. Silence allows me to observe the views of other students and learn from them or find better ways to express them. Sometimes, the classroom atmosphere is tense, and I choose silence to reduce the psychological pressure of speaking. In order to maintain a sense of security, one should be more focused on listening and thinking. On controversial topics, silence allows me to avoid disagreement or conflict with others and maintain the harmony of interpersonal relationships. When I benefit from silence in class, I will continue to keep it” (F04). “When the teacher raises a question that I am interested in, silence makes me miss the opportunity of interaction and expression in class, and I cannot exercise my expressive ability by speaking. Classroom silence also makes teachers and classmates think that I lack interest or ability, forming a negative evaluation that I do not actively participate in classroom learning. What’s worse is that the long silence makes me afraid of speaking, even doubt my ability, and lose self-confidence in learning. In the future, I will try to overcome the fear of speaking in class and gradually build up my self-confidence to speak actively” (F02).

### 4.2 Generation mechanism model of classroom silence under triadic interaction theory

**Figure 1:**
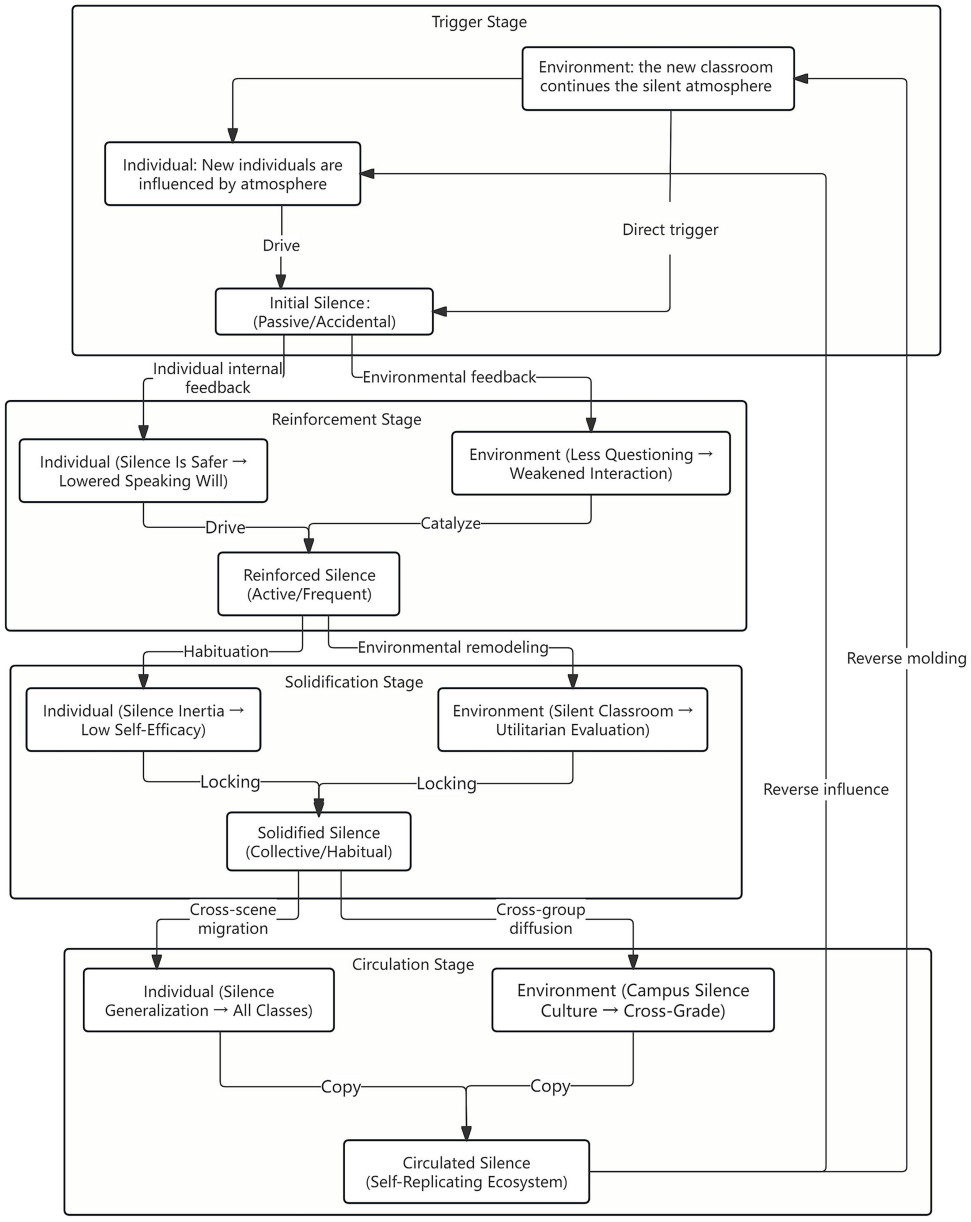
The generation mechanism model of classroom silence under triadic interaction theory.

This study attempts to construct a generative mechanism model of classroom silence under triadic interaction theory, the core of which is the “dynamic cycle.” From the “initial appearance” stage to the “solidification as normality” stage, classroom silence goes through four interconnected stages, namely, the trigger stage, reinforcement stage, solidification stage, and cycle stage, each of which reflects the three-way “individual–environmental-behavioral” interaction. The specific model (see Figure 1) is presented below.

The first stage is the triggering stage, in which individual factors interact with environmental factors for the first time and trigger the first silent behavior. At this time, classroom silence is a “situational response”; the individual has not formed a stable cognition, and it has not been solidified. If the environment is adjusted in time (such as by teachers reducing the difficulty of the problem and encouraging speech), then it can be quickly reversed. The generation mechanism of the trigger stage can be shown as two paths—one is environment → individual → behavior. For example, teachers raise questions (environment) → students speak anxiously and follow the crowd (individual) → choose passive silence (behavior). The second is individual → environment → behavior. For example, students with low self-efficacy (individuals) perceive the classroom atmosphere (environment) of “laughing at the speaker” in the class → deliberately avoid speaking and choose active silence (behavior).

The second stage is the reinforcement stage; after the initial silence behavior, through the two-way interaction of “individual reinforcement” and “environmental reinforcement,” silencing changes from “situational” to “tendentious.” Individual-environmental-behavioral forms an initial closed loop, silence changes from “accidental” to “normal,” and individual willingness to speak continues to decline. The generation mechanism of the reinforcement stage can be shown as two paths—the first is behavior → individual → behavior. For example, passive silence is not criticized by teachers (behavior) → students obtain the positive experience of “avoiding negative evaluation” and enhance the psychological state of “silence is safer” (individual) → the next time they are more inclined to silence (behavior). The second is behavior → environment → behavior. Many students are silent (behavior) → teachers reduce the frequency of questioning, and classroom interaction opportunities are reduced (environment) → more students choose silence (behavior) because of “no chance” or “conformity.”

The third stage is the solidification stage. After long-term reinforcement, silent behavior is internalized into individual habits and externalized into the characteristics of the classroom environment. The three are deeply bound and coupled to form “silence inertia.” Silence has become the “default behavior” in the classroom; even if the individual has the intention to speak, he or she will choose to obey due to the “pressure to break the silence, and the environment and individual form a “mutual lock.” The formation mechanism of the solidification stage can be divided into two paths—the first is individual → behavior → environment → individual. For example, because of long-term silence, students’ self-efficacy for speaking falls to a low ebb (individual) → actively avoid all speaking opportunities (behavior) → the class forms a “silent classroom” atmosphere (environment) → students choose silence under the influence of the atmosphere (individual). The second is environment → individual → behavior → environment. Teachers’ negative feedback on speaking (environment) → students’ fear of speaking (individual) → continuous silence (behavior) → teachers abandon interactive design and adopt “cramming” teaching (environment).

The fourth stage is the cycle stage, in which the solidified silence forms a “self-replicating” ecosystem, and the Individual-environmental-behavioral interaction no longer needs an external trigger but rather internal cycle reinforcement and even spreads to other classes and grades. Silence has been upgraded from a “single-classroom phenomenon” to “campus local ecology.” The generation mechanism of the loop phase can be represented by two paths. The first is cross-classroom diffusion, that is, behavior → individual → environment. For example, the habit of silence (behavior) formed by students in course A → transferred to course B (individual) → the environment of course B is also gradually silent (environment); the second is cross-grade diffusion, that is, behavior → environment → individual. For example, the silent behavior of senior students (behavior) becomes the “model” of junior students (environment) and the formation of the silent tendency of junior students (individual).

## 5 Discussion and analysis

### 5.1 Key advantages and application value of the classroom silence generation mechanism model under ternary interaction theory

The model deeply reveals the dynamic process of ternary interaction, including four stages of triggering, reinforcement, solidification, and circulation, and identifies the self-reinforcement mechanism of silent behavior—individual silence → group silence → environmental change → more silence, forming a vicious circle. The key advantages of this model lie in three aspects. First, it focuses on the dynamic nature of classroom silence. The model breaks through the list of static factors and focuses on “stage evolution” and “two-way feedback” to explain the process of the silence from “accidental” to “normal”; the second is to reflect the systematic nature of classroom silence. It emphasizes that the individual–environmental–behavioral interaction is not an isolated role but forms an ecosystem of “pulling the trigger and moving the whole body, avoiding a single dimension of attribution.” Third, it emphasizes the intervention of classroom silence. At each stage, the core interaction path is defined to provide targets for precise intervention. The model has important application value, which is embodied in three aspects. The first is the interpretation level. The model clearly answers the question of “why classroom silence becomes increasingly intense” and reveals the “self-reinforcing” logic of silence. The second is the diagnostic level. The model helps teachers judge the stage of classroom silence and formulate targeted strategies. The third is the intervention level. The model clarifies the focus of intervention at different stages. In the trigger stage, we need to “break the initial silence quickly” (such as via a low threshold questioning session); in the reinforcement stage, we need to “block feedback reinforcement” (such as not ignoring silence and encouraging speech); and in the solidification stage, we need “three-dimensional synchronization” (individual effectiveness improvement + environmental optimization + behavior activation). The cycle stage needs “cross-scene collaborative intervention” (such as campus culture + classroom teaching + evaluation reform).

### 5.2 The solution to classroom silence under ternary interaction theory

In accordance with the generation mechanism model of classroom silence, the following three aspects are needed to address the phenomenon of college students’ curriculum silence. The first goal is to stimulate students’ psychological system and shape more diverse classroom participation. To improve individual classroom silence, students need to reflect on their own life course, rebuild the meaning of life, and understand themselves more clearly (Wang and Wu, 2024). Individuals understand their own characteristics in terms of knowledge, ability, personality, and other aspects; explore their own interests; choose their favorite majors and courses; actively respond to the psychological barriers faced by classroom participation; enter the classroom with problems; and experience high-quality classroom interaction, in-depth learning, positive classroom thinking, etc. The second goal is to enhance the overall quality of teachers and build a more harmonious community of teachers and students. Teachers are important factors that affect classroom silence, and teachers’ understanding of and support for students is key to overcoming classroom silence (Chen, 2023). Teachers should strive to recognize, tolerate, and understand the various ways in which students participate in classrooms and establish a broader concept of participation that includes a variety of ways. In accordance with physical conditions, we should formulate corresponding teaching strategies, form diversified teaching styles, and constantly improve teaching ability. Moreover, teachers should pay attention to all students in classroom teaching, carefully design teaching scenarios, make good use of artistic language and emotional expression methods in teacher–student interactions, develop more harmonious relationships between teachers and students, and create a learning community that is full of trust and security. Creating More Democratic Classroom Practices. An equal peer relationship has a greater role in promoting students’ classroom participation, and students are more likely to have full opportunities to imitate, show, question, communicate, compete, mediate, and cooperate (Wu, 1998). A democratic classroom should be constructed in which students compete and cooperate benignly, and the learning mode of cooperative inquiry should be advocated. Through group or team cooperation, joint inquiry and other learning methods, in the process of applying knowledge and sharing opinions, we should effectively promote interactions between students, fully mobilize the enthusiasm of students for learning, and construct “learner-centered” flat classroom management so that every student can feel respected and “seen” and constantly improve students’ participation in the classroom.

## 6 Research conclusions

Classroom silence is widespread in all kinds of schools at all levels in China, and it becomes increasingly serious with increasing age. The mechanism of classroom silence needs to be explored urgently, and it has become an important topic in theoretical circles. From the new perspective of triadic interaction theory, this study comprehensively interprets the generation mechanism of college students’ classroom silence. This study revealed that the core elements of the generation mechanism of classroom silence include three dimensions. Individual factors include self-efficacy, silence motivation, and psychological status; environmental factors include teacher factors, peer factors, and collectivist norms; and behavioral factors include silent self-reinforcement, silent environmental adaptation, and silent behavior selection. In this study, a model of the classroom silence generation mechanism is constructed under ternary interaction theory, which better interprets the dynamic and systematic characteristics of the curriculum silence generation mechanism. Interventional silence involves four stages, namely, the triggering stage, strengthening stage, solidifying stage, and circulating stage, each of which reflects the two-way interaction of the dynamic “individual–environmental–behavioral” cycle.

## 7 Contributions and limitations of the study

The contribution of this study lies in both theory and practice. One is the theoretical contribution. On the one hand, from the new perspective of triadic interaction theory, in this study, a theoretical model of the generation mechanism of college students’ classroom silence is constructed, which reveals the dynamic interactive nature of classroom silence and explains the generation mechanism of college students’ classroom silence from the three core dimensions of individual, environment, and behavior. This work interprets the vicious dynamic “individual–environmental–behavioral” cycle from four interconnected stages and supplements the theoretical research results concerning the generation of classroom silence. Second, this study makes practical contributions. The results of this study can be applied to teaching practices in colleges and universities, which not only help colleges and universities alleviate the phenomenon of silence in the classroom and improve the quality of teaching and the effect of personnel training but also help college teachers effectively improve the overall quality of teaching and stimulate the vitality of the classroom and learn in depth in the classroom to achieve professional growth and self-improvement.

The limitations of this study are as follows. First, this study is a cross-sectional study; owing to the limitations of cross-sectional research methods, only through interviews at a certain time point of the research sample to obtain first-hand data some degree of emotional oversight or memory selection may occur, which may not be able to completely restore the original appearance of the generation mechanism of classroom silence. Future research could collect more data sources and research samples at different time points to circumvent the limitations of cross-sectional studies. Second, although college students grow up in the cultural context of Chinese society, they have different cultural perceptions, value identities, and growth experiences at the individual level. This study may not be able to objectively, accurately, and exhaustively describe the rich and diverse learning characteristics of Chinese college students in the new era. Future research can study the complex mechanism of classroom silence more comprehensively through a large-scale national survey of college students’ academic performance.

## Data Availability

The original contributions presented in the study are included in the article/supplementary material, further inquiries can be directed to the corresponding author.
